# Is Qualitative Research Second Class Science? A Quantitative Longitudinal Examination of Qualitative Research in Medical Journals

**DOI:** 10.1371/journal.pone.0016937

**Published:** 2011-02-24

**Authors:** Kerem Shuval, Karen Harker, Bahman Roudsari, Nora E. Groce, Britain Mills, Zoveen Siddiqi, Aviv Shachak

**Affiliations:** 1 School of Public Health, Division of Epidemiology, Human Genetics and Environmental Sciences, University of Texas at Dallas, Dallas, Texas, United States of America; 2 University of Texas Southwestern Medical Center, Dallas, Texas, United States of America; 3 The Leonard Cheshire Disability and Inclusive Development Centre, Department of Epidemiology and Public Health, University College London, London, United Kingdom; 4 Faculty of Information and Department of Health Policy Management and Evaluation, University of Toronto, Toronto, Canada; Copenhagen University Hospital Gentofte, Denmark

## Abstract

**Background:**

Qualitative research appears to be gaining acceptability in medical journals. Yet, little is actually known about the proportion of qualitative research and factors affecting its publication. This study describes the proportion of qualitative research over a 10 year period and correlates associated with its publication.

**Design:**

A quantitative longitudinal examination of the proportion of original qualitative research in 67 journals of general medicine during a 10 year period (1998–2007). The proportion of qualitative research was determined by dividing original qualitative studies published (numerator) by all original research articles published (denominator). We used a generalized estimating equations approach to assess the longitudinal association between the proportion of qualitative studies and independent variables (i.e. journals' country of publication and impact factor; editorial/methodological papers discussing qualitative research; and specific journal guidelines pertaining to qualitative research).

**Findings:**

A 2.9% absolute increase and 3.4-fold relative increase in qualitative research publications occurred over a 10 year period (1.2% in 1998 vs. 4.1% in 2007). The proportion of original qualitative research was independently and significantly associated with the publication of editorial/methodological papers in the journal (b = 3.688, P = 0.012); and with qualitative research specifically mentioned in guidelines for authors (b = 6.847, P<0.001). Additionally, a higher proportion of qualitative research was associated only with journals published in the UK in comparison to other countries, yet with borderline statistical significance (b = 1.776, P = 0.075). The journals' impact factor was not associated with the publication of qualitative research.

**Conclusions:**

Despite an increase in the proportion of qualitative research in medical journals over a 10 year period, the proportion remains low. Journals' policies pertaining to qualitative research, as expressed by the appearance of specific guidelines and editorials/methodological papers on the subject, are independently associated with the publication of original qualitative research; irrespective of the journals' impact factor.

## Introduction

Medical research has been predominantly quantitative, with randomized controlled trials (RCTs) being the gold standard of medical research, and systematic reviews of RCTs considered the highest level of evidence [1], [2]. Qualitative research, in comparison, is often not included in widely accepted classifications of evidence (e.g. SORT- Strength of Recommendation Taxonomy), or is considered the lowest level of evidence, alongside case-reports, expert opinion, and anecdotal findings [1], [3]. However, in the past decade there appears to be a growing recognition that qualitative research is well suited for the field of medicine through the development of concepts and the ability to enhance the understanding of clinicians' and patients' behavior in their “natural environment” (i.e. naturalism). Thus qualitative research may be used for assessing practitioners' and patients' attitudes, beliefs, preferences, and behaviors, and how these change over time [4]. Qualitative research enables determining ‘how’ and ‘why’ evidence is translated into clinical practice, for example, in comparison to ‘what’ evidence is translated into practice as derived from quantitative research [5], [6]. Qualitative research is especially useful for the discovery and explanation of a phenomena, as opposed to a randomized controlled trial evaluating the efficacy of an intervention, for example [7]. It may also be utilized in conjunction with quantitative studies to enhance the validity of the findings through triangulation [8], [9].

It appears that qualitative research has been gaining acceptability in the medical literature, with methodological papers on appraising the quality of qualitative research appearing in top tier journals (e.g. JAMA, Lancet) [10], [11]. Yet, even though studies have reported a substantial increase in qualitative research [12], scant empirical evidence exists supporting this statement. In fact, the proportion of qualitative research in the medical research, its growth over time, and whether and to what extent do qualitative studies actually make their way up to top tier journals or remain ‘second class research’ has rarely been investigated. Research is also lacking on factors affecting the publication of qualitative research, such as journals' policies and impact factor. To address this gap in the literature, the primary aims of the present study are twofold: (1) To describe the prevalence of qualitative papers in the medical literature over a 10 year period in journals of general medicine; and (2) To examine the association between the proportion of qualitative publications in these journals to independent variables pertaining to the journals' characteristics, i.e. impact factor, country of publication, and appearance of editorial and methodological papers on qualitative research, as well as the journals' aims and instructions.

## Methods

We conducted a quantitative longitudinal study to examine the proportion of qualitative research in medical journals over a 10 year period (1998–2007). We extracted a list of medical journal categories from the ISI Web of Knowledge Journal Citation Reports (JCR) [13]. These categories include: (1) Medical Ethics; (2) Medical Informatics; (3) Medical Laboratory; (4) Medical Legal; (5) Medicine, Research, Experimental; and (6) General and Internal Medicine. We selected the journal category of General and Internal Medicine because of its higher applicability to both qualitative and quantitative research methodologies [14]. This grouping category includes 100 journals from medical specialties such as general medicine, family medicine, internal medicine, clinical physiology, pain management, and hospital medicine. Of these journals, 33 titles were excluded based on the following criteria (see appendix S1): journals not published in English, primary focus on systematic reviews or reviews (with no original research), basic sciences (laboratory or environmental medicine), health policy and clinical guidelines focus (no original research), and core medical statistics journals. Thus, we included 67 journals of general and internal medicine published in English from 1998 to 2007. Nine of these journals were published or indexed only for a portion of the 10 year period, and were included for the period they were available (appendix S1). In addition, 5 publications changed titles during the 10 year period, and were followed under the new title name.

### Search Strategy and Qualitative Definition

We used Ovid interface of MEDLINE to search for all original research articles from each of the selected journals published from 1998–2007. To determine the proportion of qualitative research of all original research published in these journals during this 10 year period, we excluded in our search from both the numerator (i.e. original qualitative research) and denominator (i.e. all original research) systematic reviews (or reviews), comments, letters, editorials, errata, notices of retractions, and technical reports. We searched for original qualitative research studies (English language) in the 67 journals (described above) from 1998 to 2007, utilizing the Ovid interface to MEDLINE clinical queries filter (qualitative-optimized). This clinical query filter, developed by the McMaster University Health Information Resources Unit, has a specific search strategy for qualitative studies, with previously reported sensitivity and specificity of 92% [15]. Studies were included if they met the following definition of qualitative research either as the sole focus of the study or in combination with a quantitative design in the same study (i.e. mixed methods) [16]. Qualitative research was defined as a form of empirical inquiry that usually entails: (1) purposeful sampling for information-rich cases; (2) in-depth and open-ended interviews, participant/field observations, and/or document or artifact study; (3) unit of analysis are ideas, thoughts, concepts, phrases etc.; (4) methods of analysis are inductive or a combination of inductive and deductive approaches; (5) common methodological approaches include grounded theory, action research, ethnography; and (6) prevalent underlying theories include interactionism, phenomenology, and critical theory [16]–[20].

The search from the Qualitative Research Optimized Filter gleaned 8,050 qualitative articles. Two authors (KH, ZS) independently reviewed the titles and abstracts (and full text when uncertain) of all these articles to determine the inclusion of qualitative research articles based on the definition above. Of the 8,050 studies, 1,037 were selected by both evaluators, while 344 were selected uniquely. Thus, the inter-rater reliability was high (Cohen's Kappa = 0.832; 95%CI 0.803, 0.839) [21]. The remaining 344 articles were re-examined by the 2 authors (KH, ZS) jointly with a third author (KS), and deliberation was held until reaching consensus. The final set of included qualitative research articles consisted of 1,255 papers.

### Measures and Statistical Analysis

The primary dependent variable was defined as the proportion of qualitative research studies in the medical literature. The dependent variable was determined by dividing original qualitative studies published in each of the journals of general and internal medicine (numerator) by all original research articles published in that journal (denominator) for each year (1998–2007). Independent variables, potentially associated with the publication of qualitative research, consisted of: (1) Appearance of editorials and/or methodological papers on qualitative research (excluding original research) in the journal during the 10 year period (yes/no). This variable was chosen as a potential indicator of the journals' interest in publishing original qualitative research. Editorial and methodological papers were manually searched for and determined from the MEDLINE clinical queries search; (2) The journals' policy regarding qualitative research- the aims and guidelines for authors were examined from each of the journals' websites to ascertain the presence of specific guidelines for authors pertaining to qualitative research and/or specific mention of qualitative research in the journals' aims or scope. The variable was dichotomized into: Yes (qualitative research mentioned in either aims or instructions or both); No (qualitative research neither mentioned in aims nor instructions); (3) The journals' impact factor (continuous) - was derived for each year of the 10 year period from the ISI web of science Journal Citation Reports [13]; (4) Country of publication, as derived from each of the journals' websites, was categorized into 3 categories: US, UK, and all other countries (defined as ‘other’). The ‘other’ category was not divided into sub-categories to facilitate statistical analysis-due to the relatively small number of journals (n = 67).

The unit of analysis was the individual journal. For the purpose of describing the secular trend of publication of qualitative research (aim 1) we examined the proportion of qualitative research in the journals over a 10 year period (1998–2007), and also stratified by journals' country of publication. For aim 2, we assessed the association between the publication of qualitative research over the 10 year period and independent variables (i.e. country of publication, editorial/methodological papers, aim/guidelines, and impact factor). To evaluate the longitudinal association between the proportion of qualitative studies and the independent variables (mentioned above) a generalized estimating equations (GEE) approach with exchangeable correlation matrix was utilized [22]. Since there was a significant correlation between impact factor in 2007 and the impact factor in previous years, we used the 2007 impact factor in the model. Including impact factor as a time-dependent covariate in the model did not change the results materially. Data were analyzed using Stata SE 11.0 (StataCorp, College Station, TX).

## Results

**Figure 1:**
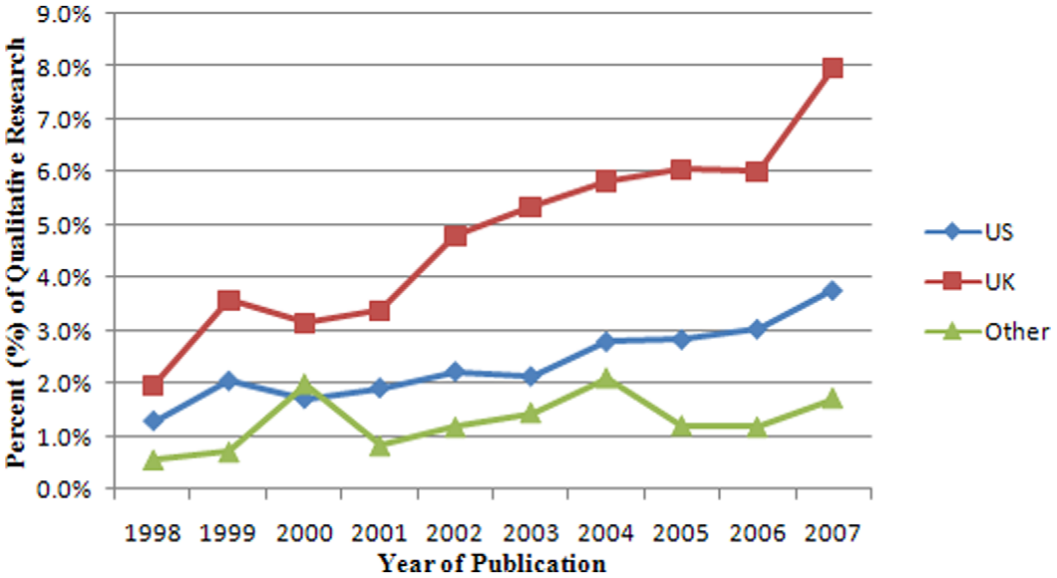
The mean percent of original qualitative research published in journals of general and internal medicine (1998–2007).

**Figure 2:**
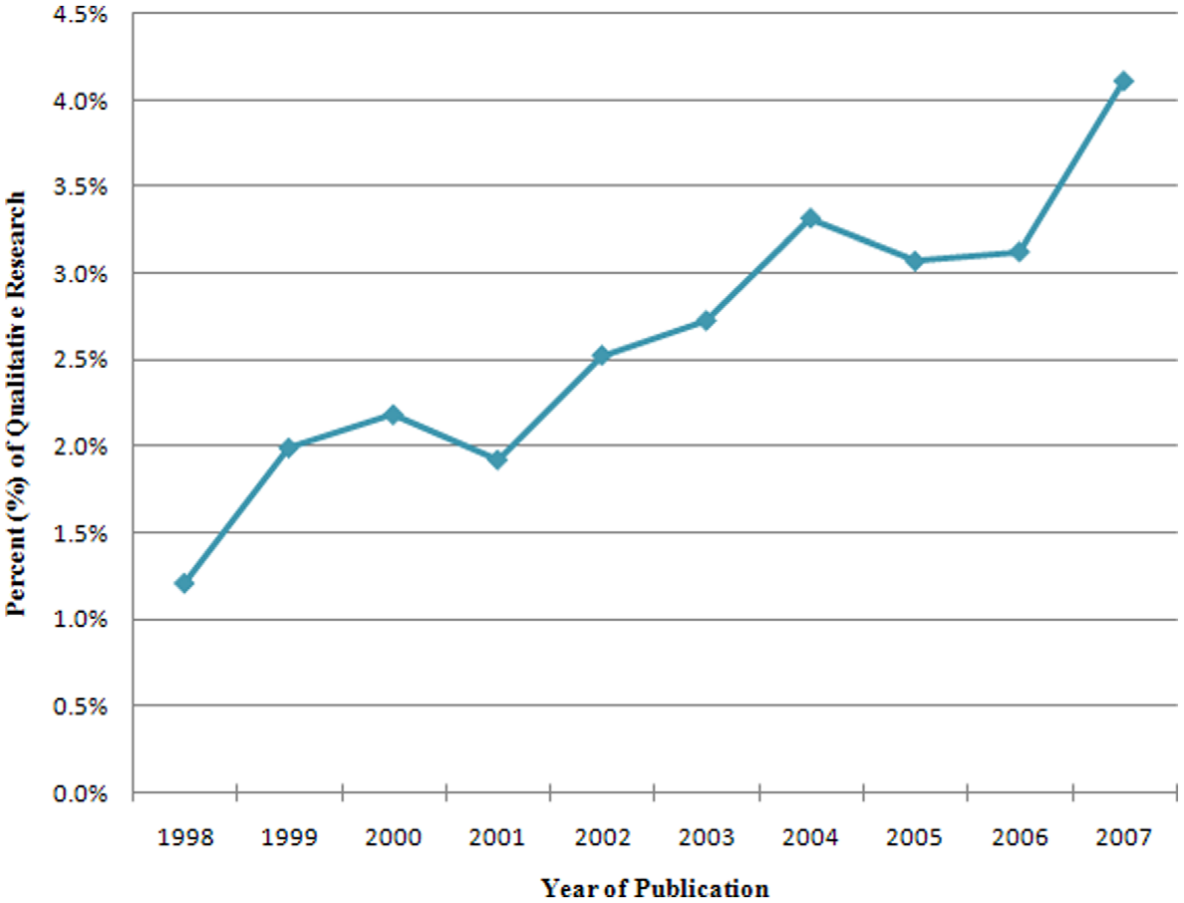
The mean percent of original qualitative research published in journals of general and internal medicine (1998–2007) - stratified by country of publication.

The proportion of original qualitative research in journals of general medicine is low, despite a steady growth over a 10 year period. In 1998, only 1.2% (SD = 2.8%) of original studies were qualitative, in 2002- 2.5% (SD 4.5%), whereas 5 years later, 4.1% (SD = 7.8%) were qualitative (figure 1). Thus, a 2.9% absolute increase and 3.4-fold relative increase in qualitative research publications occurred over a 10 year period. When examining the proportion of qualitative research in 1998 stratified by the journals' country of publication, UK journals published a higher proportion of qualitative research (mean = 7.9%, SD = 12.2%) than US journals (mean = 3.7%, SD = 5.8%) and journals published in other countries (mean = 1.7%, SD = 4.0%; figure 2). The absolute increase in qualitative publications over a 10 year period (1998–2007) was 6.0% in UK journals, 2.5% in US journals, and 1.2% in journals published elsewhere. The relative increase in UK journals (4-fold) was higher than both US journals (2.9-fold) and journals published elsewhere (3-fold).

The proportion of original qualitative research in journals of general and internal medicine was independently and significantly associated with the journals' aims and guidelines for authors, and appearance of methodological/editorial papers on qualitative research in the journal (table 1). Specifically, the presence of aims/guidelines for publishing qualitative research was the most significant predictor of actual publication of original qualitative studies, with a 6.8% increase in the probability of publishing (as compared to the absence of aim/guidelines), after adjustment for other covariates (b = 6.847, 95% CI: 4.4, 9.3%). The presence of methodological/editorial papers was associated with a 3.7% higher probability of publishing qualitative research than its absence (b = 3.68, 95% CI: 0.82, 6.5%), while controlling for covariates. Additionally, the association between country of publication and the proportion of qualitative research was of borderline significance. Thus, UK journals, on average had a 2% higher probability of publishing qualitative research during the 10 year period in comparison to other countries (b = 1.77, 95% CI: −0.18, 3.7%), while controlling for covariates. The journals' impact factor, in contrast, was not independently and statistically associated with the publication of qualitative research (table 1).

**Table 1:** Generalized Estimating Equations (GEE) Model of the Association between the Proportion of Qualitative Research and Factors Pertaining to Journals' Characteristics and Policies.

Independent variable	Proportion of Qualitative Research^1^			
	b	SE	95% CI	P-value
Country of Publication^2^				
UK	1.776	0.998	−0.180, 3.733	0.075
US	−0.306	0.943	−2.155, 1.541	0.745
Editorials/Methodological Papers (presence/absence)^3^	3.688	1.462	0.822, 6.554	0.012*
Aims/Guidelines (presence/absence)^4^	6.847	1.235	4.425, 9.268	0.000*
Impact Factor (continuous)	−0.053	0.051	−0.154, 0.047	0.298

b- Unstandardized coefficient; SE- Standard Error; CI- Confidence Interval.

*Statistically significant at the 0.05 level.

1 The proportion of qualitative research was calculated by dividing original qualitative studies published in journals of general and internal medicine from 1998 to 2007 (numerator) by all original research articles published in journals from 1998 to2007 (denominator). The proportion was calculated for each journal (unit of analysis) and for each year.

2 Country of publication- as derived from journals' website- was categorized into 3 categories: US, UK, and all other countries (defined as ‘other’). The ‘other’ category was not divided into sub-categories to facilitate statistical analysis-due to the relatively small number of journals (n = 67). The UK and US category were each compared to the referent category- ‘other’.

3 Editorial/Methodological papers- defined as appearance of editorials and/or methodological papers on qualitative research (excluding original research) during the 10 year period (1998–2007). The presence of editorial/methodological was compared to the referent category- absence of editorial/methodological papers.

4 Aims/guidelines- defined as the presence of specific guidelines for authors pertaining to qualitative research and/or specific mention of qualitative research in the journals' aims or scope. The presence of aims/guidelines was compared to the referent category -absence of aims/guidelines.

5 Impact factor – impact factor was derived for each year of the 10 year period (1998–2007) from the ISI web of science Journal Citation Reports.^10^ Since the 2007 impact factor was highly and significantly correlated with the 10-year mean (r = 09.37; P = 0.01), the 2007 impact factor was utilized.

## Discussion

To our knowledge, this is the first study to quantitatively examine the proportion of qualitative research in journals of general medicine over a 10 year period. Our results suggest that although there appears to be growing interest in qualitative research, the actual publication of original qualitative studies remains low [6], [23]. Hence, despite a 3.4-fold increase in qualitative publications over a 10 year period, only 4.1% of research was qualitative in 2007. This low proportion of qualitative research may inhibit comprehensively addressing important questions pertaining to general medicine, such as understanding patient-doctor communication, patients' medication compliance and treatment decisions, dissemination of evidence into clinical practice, or physicians' utilization of electronic medical records in complex health care settings [24]–[27]. Furthermore, the dominance of quantitative research may hinder gaining insight into how to improve health care services or delivery of care, or understand the effects of interventions as experienced by health care providers and patients [8].

Results from the present study indicate that the journals' policies regarding publication of qualitative research, as reflected by specific reference or guidelines pertaining to qualitative research or appearance of methodological papers and editorials on the subject; independently predict a higher probability of publishing original qualitative research. These findings underscore the paramount role journals' policies play in determining the proportion of qualitative research in journals of general medicine, irrespective of other variables, such as the journals' impact factor. It is difficult, however, to determine whether journals that publish methodological and editorial articles or refer to qualitative research in their policy statements are more receptive to this type of research; or alternatively whether qualitative researchers are more inclined to submit (and subsequently publish) their work in these journals because of prior publications and policy statements.

Few studies have quantitatively examined the publication of qualitative research in medical journals. McKibbon & Gadd (2004) comprehensively assessed the publication of qualitative studies in clinical journals during the year 2000 [16], in comparison to a 10-year period in our study. McKibbon & Gadd found a 3.1 times higher proportion of qualitative research than in the current study (1.86% compared to 0.6% of all articles for the same year). However, methodological differences hinder this comparison; e.g. difference in the ‘study population’ (journal type) between the current study (journals of general and internal medicine) and the McKibbon & Gadd study (wide variety of clinical journals) [16]. Petticrew et al. (2008) examined the differences in journals' acceptance rates of quantitative and qualitative studies that were previously presented in scientific conferences [28]. No significant differences were found and the authors concluded that there was no publication bias against qualitative research. However, they only tested whether a paper was eventually published in an academic journal or not. Indicators of journals' quality or characteristics were not taken into account.

An additional result stemming from the current study points to differences in the proportion of qualitative research between journals published in the UK, US, and the rest of the world; though these differences are of borderline significance. This might stem from differences in research traditions and culture between the US and Europe, with qualitative research being more common in Europe and the UK [29], [30]. Additionally, funding agencies in the UK might be more receptive and willing to fund qualitative research (resulting in more publications) than in the US. However, scant evidence exists assessing funding rates of qualitative versus quantitative submissions in both countries to support this supposition [31].

Our study has both strengths and limitations that need to be taken into account. We examined trends pertaining to the proportion of qualitative research published in 67 journals of general and internal medicine over a 10 year period. Previous studies either did not assess the proportion of qualitative studies longitudinally [16], or utilized a limited number of general medicine journals [32]. Moreover, we assessed the association between journal characteristics (as independent variables) and the proportion of qualitative research (as an outcome measure); other variables (beyond journal characteristics) are likely to impact publication of qualitative research, such as topic of research, and editorial decisions or policies not reflected in the journals' website. Since the primary objective of the study was to examine the proportion of qualitative research in the medical literature, we did not assess the quality of qualitative research (i.e. the numerator) nor did we assess the quality of the quantitative research (i.e. a component of the denominator). Moreover, we did not differentiate between mixed-methods and qualitative studies since we sought to examine the acceptability of qualitative research even when combined with a quantitative approach. The current study only focused on journals of general-internal medicine; other medical and health related disciplines were excluded. Previous research has shown that qualitative studies are more prevalent in other health-related fields, such as nursing [16]. Nonetheless, our findings are novel, and our study is the first to describe secular trends in the proportion of qualitative research in medical journals and correlates associated with its publication utilizing multivariable analysis; thereby enabling the determination of which variables are independently associated with the outcome measure. Our results suggest that even though the importance of qualitative research has been emphasized by several editorials and methodological papers in leading medical journals [10], [11], the proportion remains low. This finding indicates that medical sciences have been leaning heavily towards the quantitative view of research. Although it is hard to determine the right balance between different types of research, this dominance of the quantitative approach could potentially inhibit discovery and explanation in general medicine [8].

Results of our multivariable analysis underscore the impact journal policies have on the publication of qualitative research. For the proportion of qualitative research to continue to increase in the medical literature, more journals would need to clearly indicate that this methodology is an acceptable mode of inquiry, by providing specific reference and guidelines to authors pertaining to qualitative research. Future research should continue to monitor the proportion of qualitative research in medicine, assess whether the proportion continues to grow or stabilize, and examine the quality of published studies along with quantity. Additionally, the association between the journals' country of publication and the actual publication of qualitative research should be explored further; perhaps via interviews and/or surveys with European and American journal editors, researchers, and funding agencies to elucidate potential differences in perceptions of the acceptability of qualitative research as a legitimate method of inquiry. Interviews with journal editors and authors may also help explain the association between journal policy statements, publication of editorials on qualitative research, and the proportion of qualitative studies. These interviews may shed light on editorial practices when receiving qualitative studies and authors' choices of journals for qualitative paper submissions.

### Institutional Review Board Approval

Approval is not required since human participants are not involved.

## Supporting Information

Appendix S1 List of Journal's of General and Internal Medicine Included/Excluded and Comments.
(DOCX)
