# Supplementary material for: Is Qualitative Research Second Class Science? A Quantitative Longitudinal Examination of Qualitative Research in Medical Journals
Source: PLoS One. 2011 Feb 24;6(2):e16937. doi: 10.1371/journal.pone.0016937 (PMC3044713; doi:10.1371/journal.pone.0016937)
Supplement: Appendix S1 — List of Journal's of General and Internal Medicine Included/Excluded and Comments. (DOCX) [file pone.0016937.s001.docx]

Appendix S1: List of Journal’s of General and Internal Medicine Included/ Excluded and Comments

| Journals^1^ | Included | Excluded^2^ | Comments |
| --- | --- | --- | --- |
| 1. AM J CHINESE MED | X |  |  |
| 1. AM J MANAG CARE | X |  |  |
| 1. AM J MED | X |  |  |
| 1. AM J MED SCI | X |  |  |
| 1. AM J PREV MED | X |  |  |
| 1. ANN ACAD MED SINGAP | X |  |  |
| 1. ANN FAM MED | X |  | Started in 2003 |
| 1. ANN INTERN MED | X |  |  |
| 1. ANN MED | X |  |  |
| 1. ANN SAUDI MED | X |  | Not indexed in MEDLINE prior to 2004 |
| 1. ARCH INTERN MED | X |  |  |
| 1. BRIT J GEN PRACT | X |  |  |
| 1. BRIT MED J | X |  |  |
| 1. CAN FAM PHYSICIAN | X |  |  |
| 1. CAN MED ASSOC J | X |  |  |
| 1. CHINESE MED J-PEKING | X |  |  |
| 1. CLIN MED | X |  | Started in 2001 |
| 1. CROAT MED J | X |  |  |
| 1. CURR MED RES OPIN | X |  |  |
| 1. DAN MED BULL | X |  |  |
| 1. EUR J CLIN INVEST | X |  |  |
| 1. FAM MED | X |  |  |
| 1. FAM PRACT | X |  |  |
| 1. INDIAN J MED RES | X |  |  |
| 1. INT J CLIN PRACT | X |  |  |
| 1. INTERN MED J | X |  | Continues Aust NZ J Med |
| 1. IRISH J MED SCI | X |  |  |
| 1. ISR MED ASSOC J | X |  | Not indexed in MEDLINE between 1998 and Sept. 1999. Continues Israel Journal of Medical Sciences |
| 1. J AM BOARD FAM MED | X |  | Continues Journal of the American Board of Family Practice |
| 1. J EVAL CLIN PRACT | X |  |  |
| 1. J FAM PRACTICE | X |  |  |
| 1. J FORMOS MED ASSOC | X |  |  |
| 1. J GEN INTERN MED | X |  |  |
| 1. J INTERN MED | X |  |  |
| 1. J INVEST MED | X |  |  |
| 1. J KOREAN MED SCI | X |  |  |
| 1. J PAIN SYMPTOM MANAG | X |  |  |
| 1. J R SOC MED | X |  |  |
| 1. J TRAVEL MED | X |  | Not indexed in MEDLINE prior to March 1998 |
| 1. J URBAN HEALTH | X |  |  |
| 1. J WOMENS HEALTH | X |  | Title change to and from Journal of Women's Health and Gender-Based Medicine |
| 1. JAMA-J AM MED ASSOC | X |  |  |
| 1. LANCET | X |  |  |
| 1. MED J AUSTRALIA | X |  |  |
| 1. MED PRIN PRACT | X |  | Not indexed in MEDLINE prior to 2002 |
| 1. MIL MED | X |  |  |
| 1. MT SINAI J MED | X |  | Only one issue published in 2007. Journal underwent editorial changes. |
| 1. NATL MED J INDIA | X |  |  |
| 1. NETH J MED | X |  |  |
| 1. NEW ENGL J MED | X |  |  |
| 1. PAIN MED | X |  | Started in 2000 |
| 1. PALLIATIVE MED | X |  |  |
| 1. PANMINERVA MED | X |  |  |
| 1. PLOS MED | X |  | Started in 2004 |
| 1. POSTGRAD MED J | X |  |  |
| 1. PREV MED | X |  |  |
| 1. QJM-INT J MED | X |  |  |
| 1. SAMJ S AFR MED J | X |  |  |
| 1. SAUDI MED J | X |  | Not Indexed in Medline prior to 2000 |
| 1. SCAND J PRIM HEALTH | X |  |  |
| 1. SCOT MED J | X |  |  |
| 1. SOUTH MED J | X |  |  |
| 1. SWISS MED WKLY | X |  | Continues Schweizerische Medizinishe Wochenschrift |
| 1. TOHOKU J EXP MED | X |  |  |
| 1. TRANSL RES | X |  | Continues Journal of Laboratory and Clinical Medicine |
| 1. UPSALA J MED SCI | X |  |  |
| 1. W INDIAN MED J | X |  |  |
| 1. ACTA CLIN BELG |  | X |  |
| 1. AM FAM PHYSICIAN |  | X |  |
| 1. AMYLOID |  | X |  |
| 1. ANNU REV MED |  | X |  |
| 1. AVIAT SPACE ENVIR MD |  | X |  |
| 1. B ACAD NAT MED PARIS |  | X |  |
| 1. BRIT J HOSP MED |  | X |  |
| 1. BRIT MED BULL |  | X |  |
| 1. CLEV CLIN J MED |  | X |  |
| 1. COCHRANE DB SYST REV |  | X |  |
| 1. DEUT MED WOCHENSCHR |  | X |  |
| 1. DM-DIS MON |  | X |  |
| 1. HOSP MED |  | X |  |
| 1. INTERNIST |  | X |  |
| 1. J LAB CLIN MED |  | X |  |
| 1. MAYO CLIN PROC |  | X |  |
| 1. MED CLIN N AM |  | X |  |
| 1. MED CLIN-BARCELONA |  | X |  |
| 1. MED KLIN |  | X |  |
| 1. MED PROBL PERFORM AR |  | X |  |
| 1. MED SPORT |  | X |  |
| 1. MEDICINA-BUENOS AIRE |  | X |  |
| 1. MEDICINE |  | X |  |
| 1. PRESSE MED |  | X |  |
| 1. PRIMARY CARE |  | X |  |
| 1. PRIMARY CARE COMMUN |  | X |  |
| 1. REV CLIN ESP |  | X |  |
| 1. REV INVEST CLIN |  | X |  |
| 1. REV MED CHILE |  | X |  |
| 1. REV MED INTERNE |  | X |  |
| 1. TERAPEVT ARKH |  | X |  |
| 1. WIEN KLIN WOCHENSCHR |  | X |  |
| 1. YONSEI MED J |  | X |  |

^1^ Abbreviated journal titles were used. Journals were extracted from the ISI Web of Knowledge Journal Citation Reports (JCR) in the category of Medicine- General and Internal.^10^

^2^ Journals were excluded based on the following criteria: not published in English, primary focus on systematic reviews or reviews (with no original research), basic sciences (laboratory or environmental medicine), health policy and clinical guidelines focus (no original research), and core medical statistics journals.
